# Particle size distribution predicts particulate phosphorus removal

**DOI:** 10.1007/s13280-017-0981-z

**Published:** 2017-11-21

**Authors:** Mark River, Curtis J. Richardson

**Affiliations:** Duke University Wetland Center, Nicholas School of the Environment, Durham, NC 27708 USA

**Keywords:** Best management practices (BMP’s), Eutrophication, Nano phosphorus, Particle size distribution, Particulate phosphorus, Stormwater wetlands

## Abstract

Particulate phosphorus (PP) is often the largest component of the total phosphorus (P) load in stormwater. Fine-resolution measurement of particle sizes allows us to investigate the mechanisms behind the removal of PP in stormwater wetlands, since the diameter of particles influences the settling velocity and the amount of sorbed P on a particle. In this paper, we present a novel method to estimate PP, where we measure and count individual particles in stormwater and use the total surface area as a proxy for PP. Our results show a strong relationship between total particle surface area and PP, which we use to put forth a simple mechanistic model of PP removal via gravitational settling of individual mineral particles, based on a continuous particle size distribution. This information can help improve the design of stormwater Best management practices to reduce PP loading in both urban and agricultural watersheds.

**Electronic supplementary material:**

The online version of this article (10.1007/s13280-017-0981-z) contains supplementary material, which is available to authorized users.

## Introduction

Humans have more than doubled the inputs of phosphorus (P) to freshwater, leading to worldwide eutrophication of water resources which impacts aquatic ecosystems and drinking water supplies (Carpenter 2008). In many watersheds, the vast majority of annual P loading occurs during storms, with much of this delivery consisting of particulate phosphorus (PP) (Duan et al. 2012; Janke et al. 2014). Phosphate is one of the strongest adsorbing anions (Schlesinger and Bernhardt 2013), and in highly-weathered landscapes such as the Piedmont, much of this P is sorbed to the outside of small mineral particles and therefore transported along with the sediment during stormflow. Small suspended sediments in particular can be enriched in P due to the preferential erosion and mobilization of fine particles from surface soils (Massey and Jackson 1952; Sharpley 1980).

In order to intercept runoff and settle out suspended sediment and associated PP, stormwater wetlands are often prescribed as a Best management practice (BMP) in urban and agricultural watersheds. However, the sizing of these stormwater basins is often based on empirical relationships derived from regression analysis of a large data cloud, resulting in performance uncertainty (Kadlec and Wallace 2008). The effectiveness of BMP’s targeted to reduce sediment erosion and associated P delivery can be greatly affected by particle size distribution (Bäckström 2003; White et al. 2007), with clay particles often not removed effectively (Deletic 1999). A better understanding of the behavior of particles could help us to improve stormwater BMP designs and reduce PP loading to downstream aquatic ecosystems.

This study addresses the following research questions:

- What is the relationship between PP and the total normalized surface area of particles, as measured via flow-imaging particle size analysis?
- Can the removal of PP via quiescent settling be predicted with a continuous particle size distribution obtained via flow-imaging particle size analysis?
- How does a mechanistic model of PP removal based on continuous particle size distribution differ from first-order decay models of P removal?

## Background

### Relationship between particles and P

Public data from the United States Geologic Survey (USGS) show that many watersheds have a very strong relationship between total suspended solids (TSS) and total P. However, while being a good predictor of P, TSS ultimately gives little insight into the mechanisms of PP removal via BMP’s, since it is a lumped parameter without any detailed information about particle size distributions (Gao 2008).

Particles have been classified as sand, silt, or clay for nearly a century (Wentworth 1922). However, over the last several decades, researchers have increasingly emphasized the importance of finer-resolution particle size distributions for accurate modeling of processes affecting water quality (Sheldon et al. 1972; Pearson 1985). The lack of accurate, high-resolution particle measurements has hindered the mechanistic modeling of PP removal from stormwater. Fortunately, recent advances in flow-imaging particle size analysis technology for particle measurement now allows for finer resolution and improved sensitivity. More accurate measurement of particle size distributions in stormwater thus allows for development and testing of mechanistic models of PP removal, which were not possible with earlier technologies. If PP is controlled by surface processes on particles, then surface area may be an appropriate proxy for PP.

Phosphate sorption/desorption has been shown to occur as a rapid first-order reaction followed by a slower second-order reaction (Papadopoulos et al. 1998; McDowell and Sharpley 2003). Studies have shown that sorption/desorption of soluble P can happen very rapidly during stormflow, with soil material sometimes acting as a sink and sometimes as a source, depending upon local conditions (Sharpley et al. 1981; Froelich 1988). The ability for rapid sorption/desorption of P in stormflow means that P is more likely to be spread among the particles in stormwater as a function of exchange sites, which can be approximated by surface area. Indeed, several studies have shown that P is primarily sorbed to the outer surface of particles and therefore surface area can be used as a reasonable proxy for P sorption (Al-Kanani and MacKenzie 1991; Wang et al. 2001), particularly if phosphate reaches an equilibrium partitioning across the surface area distribution of the entire particle size range (Kim et al. 2008). This surface-bound P can either occur as phosphate sorbed to mineral particles, organic matter coatings on sediment grains (Horowitz and Elrick 1987), or phosphate bound via ligand exchange to humic-Fe complexes (Gerke and Hermann 1992). With respect to mineral particles, organic matter, and/or iron oxide coatings can fill in granular imperfections producing a more uniform surface (Pacini and Gächter 1999). For clay-sized particles, a relatively uniform coating of particle surfaces could improve the accuracy of using spherical surface area as a proxy for PP, as otherwise uncoated plate-like clay particles would be expected to have a much different relationship between P sorption and surface area than an idealized sphere (Bar-Yosef et al. 1988). Ultimately, the extent to which surface processes drive the overall P content of particles will determine whether surface area is a good proxy for PP in a given watershed (Effler et al. 2014).

### Stormwater wetlands and PP removal

Stormwater wetlands remove P via physical, chemical, and biological processes (Wong et al. 2006). For PP, removal is often dominated by the physical process of gravitational settling of particles. Stokes’ Law calculates the settling velocity of a particle as a function of its diameter squared (Stokes 1851). More recent investigations by Gibbs et al. (1971), Komar (1981), and Le Roux (1992) have demonstrated that Stokes’ theoretical settling velocities apply fairly well for small, near-spherical particles of various mineralogy in water, particularly at low Reynolds numbers.

For abiotic removal of PP via settling of particles, Stokes’ Law implies that each individual size class of particle settles out at a different velocity, and therefore has its own individual removal rate. However, many previous studies have used either a lumped effective settling velocity for the entire particle size range of stormwater (Carleton et al. 2001), or have classified particles into just a few size classes to describe the behavior of PP (Lee et al. 1989; Arias et al. 2013). Unfortunately, lumping the entire distribution of particle sizes into one settling velocity or into just a few categories ignores the intricacies of particle removal; at steady-state flow conditions, this might be a reasonable assumption (Carleton et al. 2001; Kadlec and Wallace 2008), but for pulse-flow stormwater basins it doesn’t give much insight into how to most effectively target a BMP.

In our model, we use a novel flow-imaging method to count and measure individual particles in stormwater, producing a near-continuous particle size distribution. This method, therefore, gives additional mechanistic insights regarding how these particles and associated PP could be removed in stormwater wetlands via gravitational settling.

## Materials and methods

### Study area

Our study site is the Falls Lake Watershed within the North Carolina Piedmont near Raleigh–Durham. The U.S. Piedmont (derived from the Latin *pes montium*, literally foot of the mountain) is a highly-weathered landscape, which lies between the Coastal Plain and the Appalachian Mountains stretching from New Jersey south to Alabama. Soils in the Southeastern Piedmont region tend to be very deep with long groundwater residence times (Rose and Fullagar 2005), and streams are often transport-limited resulting in much of the sediment delivered to the stream being stored as alluvium (Phillips 1991). In highly-weathered landscapes such as the Piedmont, iron tends to be conserved in soils and sediments due to its poor solubility (Megonigal et al. 2004). Iron oxides have large P binding abilities (Parfitt et al. 1975; Richardson 1985); and soils in the Southeastern Piedmont have been shown to have a large capacity to bind phosphate, largely due to their high iron content (Mayhew et al. 2001).

The North Carolina Piedmont relies almost exclusively on surface water to meet its drinking water needs. This region is predicted to undergo intense pressures in the coming decades from land use conversion from farmland/forest to suburban, and also from a water-supply standpoint driven by population growth. At the same time that the North Carolina Piedmont will be asked to provide more clean water to the region, its base of undeveloped land that provides clean water will be undergoing a transition towards increasing population and development. Conversion from farmland/forest to impervious surfaces will result in increased stormwater runoff which can mobilize and transport the highly erodible Piedmont soils and associated nutrients (Voli et al. 2013). Increasing population in the region will put pressure on the quantity of water supplied from these surface water resources. In addition, population growth in urban/suburban areas of the watershed suggests that wastewater utilities will have to service more customers or build additional facilities, while population growth in rural portions of the watershed will result in more septic tanks discharging into subsurface water resources. These cumulative impacts will pose a risk to the same water supplies that are indispensable for future economic growth.

P is a major risk to surface waters in the Piedmont and throughout the nation. The Falls Lake Watershed supplies drinking water to over 500 000 residents. Plans are currently underway to harness even more surface water as the Raleigh/Durham/Chapel Hill region continues to grow. Protecting these watersheds will be critical to sustain economic growth along with a high quality of life. As the majority of drinking water in the Piedmont comes from reservoirs, the transport of particle-bound nutrients/contaminants plays a critical role in water quality. Most PP flux in the Piedmont occurs during storms, which dominates the annual P watershed loading. In the North Carolina Piedmont, stormwater BMP’s are often utilized for new development (Gagrani et al. 2014) and as a retrofit for existing development (DeBusk et al. 2010). Stream restoration projects often include floodplain reconnection to enhance P sedimentation of PP loads during storm events (Richardson et al. 2011).

### Sample collection and PP analysis

Samples for establishing a relationship between normalized total surface area and PP were collected from various storm events in Piedmont streams along with laboratory settling tests of stormwater, for a total of 43 data points. Samples for our settling experiments were collected on two separate storm events with different particle size distributions; one event was urban stormwater from a Southern Piedmont stream, and the other event was storm runoff from an urban construction site. Total P was measured colorimetrically, following persulfate digestion, via the molybdate blue method of Murphy and Riley (1962). The same stormwater samples were also filtered through a 0.02 micron membrane filter and analyzed for total dissolved P; PP was then calculated as the difference between total P and total dissolved P. We used a smaller filter than the standard 0.45 micron, since our clay-rich watershed has a variety of naturally-occurring P-containing nanoparticles (nano phosphorus); this allows for a more accurate measurement of particulate P. Otherwise, small particles can pass through the filter and are incorrectly measured as soluble P (i.e., Hens and Merckx 2002; Filella et al. 2006).

### Particle size analysis method

**Fig. 1:**
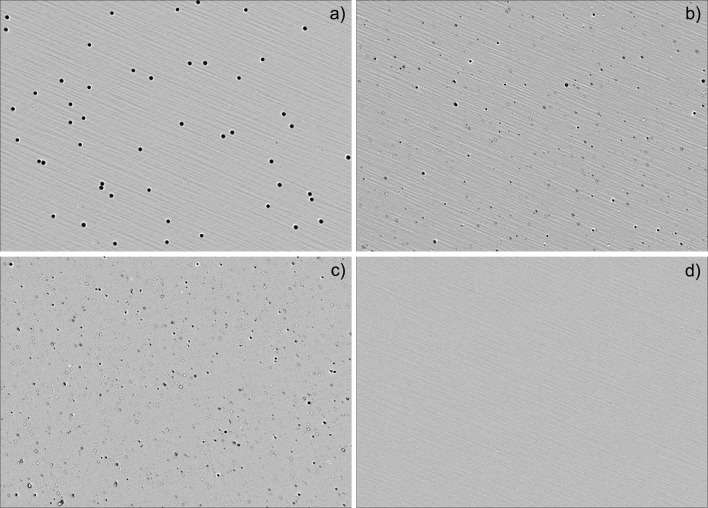
Actual screenshot of Occhio particle analysis of: **a** 4.6 μ quality control particles, **b** weak micro-ground coffee, **c** urban Piedmont stormwater, **d** urban Piedmont stormwater through 0.02 μ filter

Samples were analyzed within 4 h of collection to minimize any artifacts from post-sampling particle aggregation. Particle size analysis was performed by pumping 1 ml of stormwater sample through an Occhio Flowcell FC200S+, after gently shaking the container to re-suspend any particles which might have settled out between collection and analysis. Flow-imaging slowly draws water through a syringe pump and takes high-quality digital images at × 4.5 magnification as the water passes through a thin glass plate. For every image, an algorithm measures and counts each individual particle, and produces a particle size distribution with particle size classes down to 0.1 micron. In a typical 1 ml stormwater sample, tens of thousands of particles are counted and measured, giving robust statistics. Figure 1 shows actual screen shots from this method. For quality control, we ran a known particle size standard through the machine to focus the lens and verify its accuracy before analyzing stormwater; our results were similar to that published for other flow-imaging equipment. We also ran a sample of homogenized whole milk as a second quality control, which produced a mean particle size in line with literature values. Our particle size distributions of Piedmont stormwater are also comparable with a previous particle size analysis of Georgia kaolinite via laser scattering, which calculated a similarly shaped distribution for the smallest kaolinite fraction (Mackinnon et al. 1993).

Image analysis of suspended sediment in flowing water has several advantages and disadvantages compared to other particle size methods. Advantages include: (1) particle shading is not as much of an issue as it can be with laser-based methods, allowing broader distributions to be analyzed without separation; (2) it is not necessary to assume Mie versus Raleigh scattering nor estimate a refractive index as with some light scattering methods; (3) dispersing agents are not necessary, which could interference with settling calculations by inhibiting coagulation; (4) it is not necessary to remove organic matter, which could also affect settling dynamics; (5) low-density or neutral buoyancy particles are easily measured; and (6) it is not necessary to dry out the stormwater sample in order to analyze it (as with a scanning electron microscope), which could possibly affect particle dimensions due to shrinkage.

One disadvantage of current image-based particle size analysis of flowing water is that the lower detection limit (one pixel) is around 0.2 microns; data near this lower size range will therefore tend to be noisy. Particles smaller than this are likely either lumped into the smallest size bin, or are not counted at all. Another disadvantage is that mineral particles larger than very fine sand are difficult to accurately analyze without constant stirring of the sample, since they settle out during the several minutes required for analysis. Stirring is undesirable since it can increase coagulation compared to non-stirred samples and therefore can potentially give an inaccurate distribution. Depending upon the flow-imaging equipment used, the size of the siphon hose, and the distance between the glass plates can also limit the size of particles counted (the maximum size in our setup is approximately 100 microns). Therefore, the equipment we are using is most accurate for particle size distributions with a maximum particle size of 100 microns, and with the vast majority of particles larger than 0.2 microns. The stormwater in our watershed meets these criteria for most storm events.

### Mechanistic model of PP removal

Our simplified model is the following:

For a given particle, its distance settled as a fraction of a water column over time can be represented as:

$$ {\text{Distance}}_{\text{settled}} = \frac{{(V_{d} *t)}}{h} $$

where *V*_*d*_ is the settling velocity for particle size *d* (cm s^−1^), *t* is the settling time (s), *h* is the height of water column (cm), *Note* at time *V*_d_*t = *h*, the particle will have settled out of the water column.

Assuming *n* particles of identical size *d* are initially equally distributed throughout a water column, and are settling via plug-flow vertically through the water column; the number of particles of this size which have settled out at a given time is:

$$ {\#\text{ Particles}}_{\text{settled}} = \frac{{(V_{d} *t)}}{h}*n_{d} $$

where *n*_*d*_ is the number of particles for particle size class *d*, *Note* after time *V*_d_*t = *h*, all the particles of size class *d* will have settled out of the water column.

If each of these particles of size class *d* can be approximated as a sphere, with P sorbed to the outside of the particle, then the amount of associated PP removed over time is:

$$ {\text{PP}}_{\text{removed}} = \frac{{(V_{d} *t)}}{h}*n_{d} *A_{d} $$

where *A*_*d*_ is the surface area of particle size *d.*

Let us now consider stormwater as a mixture of different particle sizes, with PP as the summation of the surface area of trillions of individual particles. PP removed over time is, therefore, a summation of the removal of each individual particle and its associated surface area, across the entire range of particle sizes in the stormwater:

$$ {\text{PP}}_{\text{removed}} = \mathop \sum \limits_{d} \left( {\frac{{(V_{d} *t)}}{h}*n_{d} *A_{d} } \right) $$

If we insert variables for settling velocity and the surface area of a sphere, this equation can be written as:

$$ {\text{PP}}_{\text{removed}}\,=\,\mathop \sum \limits_{d} \left( {\frac{{gd^{2} \left( {\rho_{\text{p}} - \rho_{\text{m}} } \right)}}{18\mu }*\frac{t}{h}*n_{d} *4\pi *\left( {\frac{d}{2}} \right)^{2} } \right) $$

where *g* is the acceleration of gravity (cm s^−2^), *d* is the particle diameter (cm), *ρ*_p_ is the density of particle (g cm^−3^), *ρ*_m_ is the density of water (g cm^−3^), *µ* is the dynamic viscosity of water.

Here, we see the strong influence of particle diameter on PP removal, as its square drives the calculation of both settling velocity and surface area. By calculating the PP removed at time = (*h*/*V*_*d*_) for all diameters of our suspended solids, we can plot the cumulative PP removal over time. The rate of removal can then be calculated by the difference in PP removed for a given time step.

### Model assumptions

Our simplified model assumes that particle settling velocity doesn’t increase or decrease due to coagulation (e.g., Smoluchowski 1917), Brownian motion, hindered settling (e.g., Ham and Homsy 1988), or effects of vegetation. Since our model assumes a uniform distribution of suspended sediment throughout the water column prior to settling detention, we are not considering any sediment/PP transported via bedload, nor any resuspension of particles once they settle out (via wind, bioturbation, etc.,). Obviously, some of these processes can occur in natural systems depending upon numerous environmental variables, particularly for colloidal particles under high hydraulic loading rates.

X-ray diffraction data indicates that the majority of stormflow particles in our watershed are quartz, feldspar, kaolinite, illite, and smectite (Fig. S1), which have densities ranging from 2.5 to 2.8 g cm^−3^ (Parkin 1999). Our simplified model, therefore, assumes that suspended particles in our watershed can be approximated by spherical surface area with a density of 2.65 g cm^−3^, this implies that the model will be most accurate where PP loading is dominated by mineral particles as opposed to stormwater dominated by organic matter. Since organic matter has a different density than mineral particles, we would expect it to settle at a different rate. Furthermore, organic matter has been shown to increase the coagulation and settling rate of colloidal iron-containing clay particles (Pizarro et al. 1995), and therefore could also affect the settling rate of mineral particles themselves.

### Settling experiment

To mimic the conditions of particles settling out in a stormwater wetland, we conducted two separate quiescent settling experiments in the laboratory, in which we poured stormwater into fifty separate 50 ml vials with a settling depth of 10 cm. Then, in quintuplicate, we decanted the stormwater at various time steps to remove the unsettled particles from the settled particles. Particle size analyses of the decanted stormwater were conducted as soon as possible after each settling interval so as to minimize error (Phillips and Walling 1995). PP was then measured for each decanted sample, and plotted along with the values predicted by our mechanistic model.

## Results and discussion

**Fig. 2:**
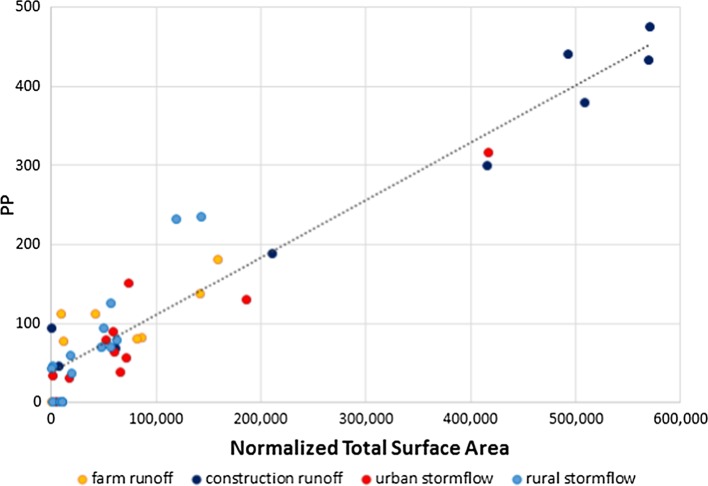
Relationship between PP (μg l^−1^) and normalized total surface area for various storm samplings and lab-settling experiments

The particle size distribution of the stormwater which we used for our first laboratory settling experiment showed a mean particle size around 0.5 microns, with the vast majority of particles under 1 micron (Fig. S2). In order to analyze the fit between PP and surface area, we conducted a linear regression of PP versus normalized surface area (SA) for different steps during the settling experiment, along with filtered samples, from a wide range of land uses in the watershed (Fig. 2). Normalized total surface area (SA) was calculated via summation of the surface area of each individual particle counted via flow-imaging particle size analysis. Most of the variance in PP can be explained by SA, providing support for the hypothesis that SA is strongly related to PP in our watershed, and that our method of calculating and summing the surface area of each individual particle is a reasonable approximation of PP content of stormwater dominated by mineral particles.

**Fig. 3:**
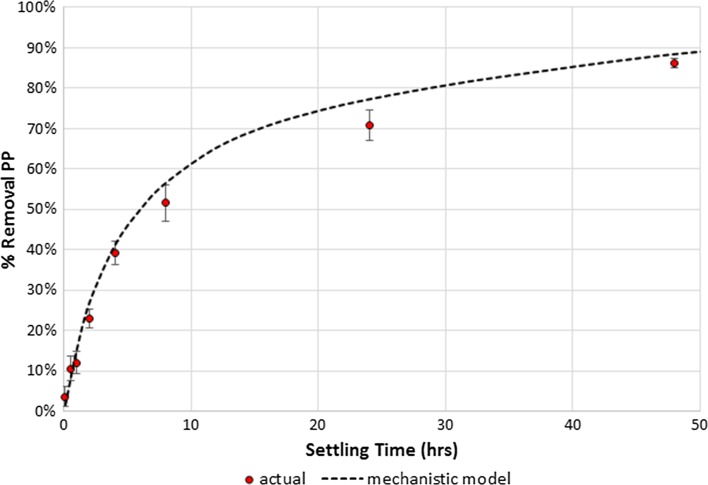
Plot of actual PP removed in lab-settling experiment of urban stormwater, compared to mechanistic model based on continuous particle size distribution of the same stormwater sample. Error bars indicate standard error of the mean of quintuplicate samples

A computer model was designed inputting the number of particles and associated surface area for each particle size class, with 100 size class bins ranging from 0.1 microns to 10 microns. PP was then partitioned evenly across the total SA. Using Stokes’ Law, the settling of these particles and their associated PP was calculated over time. These modeled results were then compared to our actual PP removal in the lab experiment (Fig. 3). Our model provides a reasonable fit to the actual settling data, which supports the hypothesis that continuous particle size distribution can be used as a tool to predict PP removal.

**Fig. 4:**
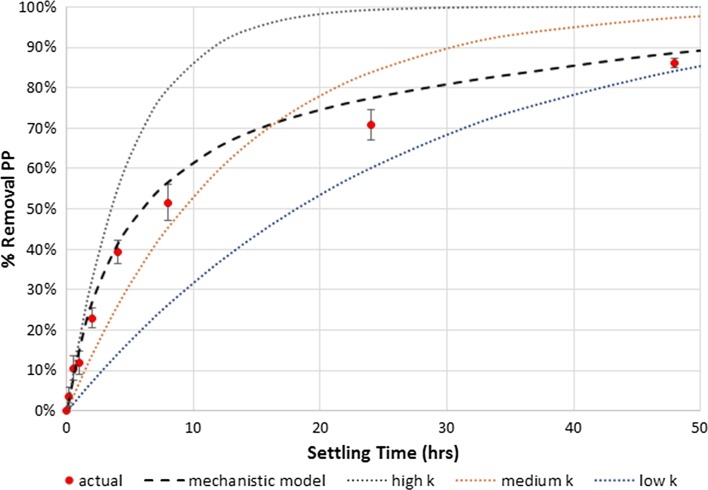
Comparison of mechanistic model of PP removal (based on measured particle size distribution) and first-order decay models with high, medium, and low k. Error bars indicate standard error of the mean of quintuplicate samples

A test for first-order dynamics by plotting ln(PP) versus time shows that PP removal dynamics are only pseudofirst-order during the very early stages of gravitational settling (when all or most of the particle size classes are settling out). As the particle size classes progressively settle out, the removal rate decreases and the overall removal deviates from first-order dynamics. This is in contrast to the commonly used first-order decay models for P removal, which assume that the rate constant k is the same throughout time. Using our model, we can also simulate the impact of different particle size distributions on the dynamics of PP removal; as particle size distributions shift towards larger particles carrying more of the P, the removal dynamics shift further from first-order with the rate constant k changing at a faster rate than with particle size distributions skewed towards smaller particles. Figure 4 shows our model results compared to traditional first-order decay models with high, medium, and low values for the decay constant k, for a lab-settling experiment of stormwater from an urban Piedmont construction site. A separate lab-settling experiment, with stormwater from an urban Piedmont stream, also showed a good mechanistic model fit compared to traditional first-order decay models (Fig. S3).

Our model simulations suggest that first-order decay models are most applicable to PP removal in the early stages of settling when all or most of the particle size classes are settling out. As larger particles within the particle size distribution begin to settle out, the dynamics deviate sharply from first-order, particularly over longer settling times. With a first-order decay model, it is difficult to predict both the early settling behavior and the late settling behavior of stormwater, since these models assume a constant rate of removal. Our mechanistic model performs better throughout the settling duration, as it accounts for a slowing rate of PP removal as larger particles settle out of solution. This insight is only possible by conceptualizing PP as the cumulative surface area of billions of individual particles, which then settles out over time at velocities dependent upon the particle size distribution. Particle size distributions skewed towards larger particles will therefore remove PP at a faster rate than distributions skewed towards smaller particles, even if both distributions have the same mass (TSS). In contrast, measuring TSS without knowledge of particle size distributions doesn’t give any insight into the rate of PP removal over time.

The mean particle size in our watershed is around 0.5 microns, with the vast majority of particles under 1 micron, this distribution was confirmed via scanning electron microscopy (Fig. S4), in addition to flow-imaging particle size analysis. Therefore, stormwater BMP’s which rely on short-term gravitational settling will only be partially effective in our watershed since they remove just a small portion of the P load due to the long settling times (days vs. hours) of these sub-micron particles. BMP’s in watersheds such as ours, with a particle size distribution skewed towards small particle sizes, should encourage long setting times and/or infiltration of stormwater (such as riparian floodplain reconnection, e.g., Richardson et al. 2011) to remove P associated with slower settling clay particles.

Our findings that normalized total surface area has a strong relationship with PP can be applied to any full-size facility, independent of settling conditions. Our experiments were performed under quiescent settling conditions, which allow for testing of our straightforward mechanistic model. We did not conduct particle settling tests under turbulent conditions, therefore, our settling experiment results might not be applicable to turbulent hydrodynamic conditions, which may prevail for some stormwater BMP’s with short stormwater retention times and which would have additional forces acting upon the particles in addition to the downward gravitational force and the upward drag force due to buoyancy. Moreover, a settling pond, often placed before wetland treatment cells will reach 60–80 % of the efficiencies of our laboratory settling columns due to flow nonidealities (Perry and Green 1984; Kadlec and Wallace 2008). However, wetland stormwater pond BMP’s should improve settling PP rates due to increased wetland retention times due to macrophyte drag exerted by dense stems and litter (Kadlec and Wallace 2008). More research is needed to test mechanistic settling models, based on particle size distributions, under field and turbulent conditions.

## Conclusions

Our mechanistic model, which used a continuous particle size distribution to approximate both the settling velocity and surface area of each individual particle, provided a good estimate of the actual PP removal dynamics of our laboratory settling experiment. The mechanistic model more accurately predicted actual PP removal over time via quiescent settling than did traditional first-order decay models.

Unfortunately, information regarding continuous particle size distributions in stormwater for watersheds of various geology and land use is lacking in the current scientific literature. As a scientific community, we should collectively move away from the mass-based measurements of percent sand/silt/clay and instead move towards more detailed measurements of particle size distributions, particularly when studying pollutants which are controlled by surface processes on particles. While the relationship between PP and bulk measures of stormwater particles (TSS, turbidity) is strong, little is known about the actual particles themselves in terms of particle size and chemistry.

High-tech tools for particle size analyses continue to improve. Fine-scale measurements of particle size can help advance our understanding of nutrient fluxes and improve the design and performance of stormwater BMP’s. We feel that there is great opportunity for research at the scale of the individual stormwater particle, which could improve our understanding of stormwater transport and ultimately lead to improvements in BMP’s at the watershed scale. Future research efforts should investigate mechanistic PP removal in plug-flow and continuous flow stormwater wetlands, where the smallest particle sizes (or flocs thereof) might not have a chance to settle out due to entrainment in slowly moving stormwater.

## Electronic supplementary material

Below is the link to the electronic supplementary material.

Supplementary material 1 (PDF 232 kb)
