# Supplementary material for: Particle size distribution predicts particulate phosphorus removal
Source: Ambio. 2017 Nov 21;47(Suppl 1):124–33. doi: 10.1007/s13280-017-0981-z (PMC5722747; doi:10.1007/s13280-017-0981-z)

Ambio

Electronic Supplementary Material

This supplementary information has not been peer reviewed.

Title: Particle Size Distribution Predicts Particulate Phosphorus Removal: a Mechanistic Model and Implications for Stormwater BMP's

Authors: Mark River, Curtis J. Richardson

Figure S1: X-ray diffraction data from a typical stormwater sample from the North Carolina Piedmont. Predominant minerals include quartz, feldspar, smectite, illite, and kaolinite; this was used to inform our modeled estimate of particle size density.

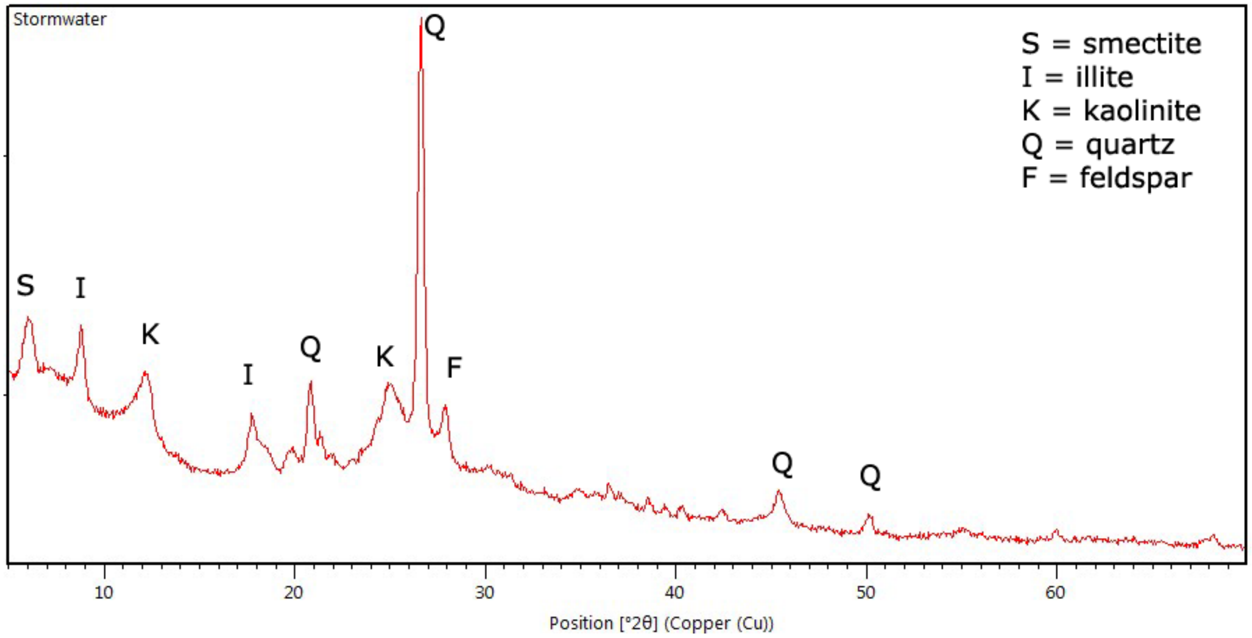

Figure S2: Particle size distribution of Piedmont stormwater, determined by pumping 1ml of sample through Occhio flow-imaging particle size analyser.

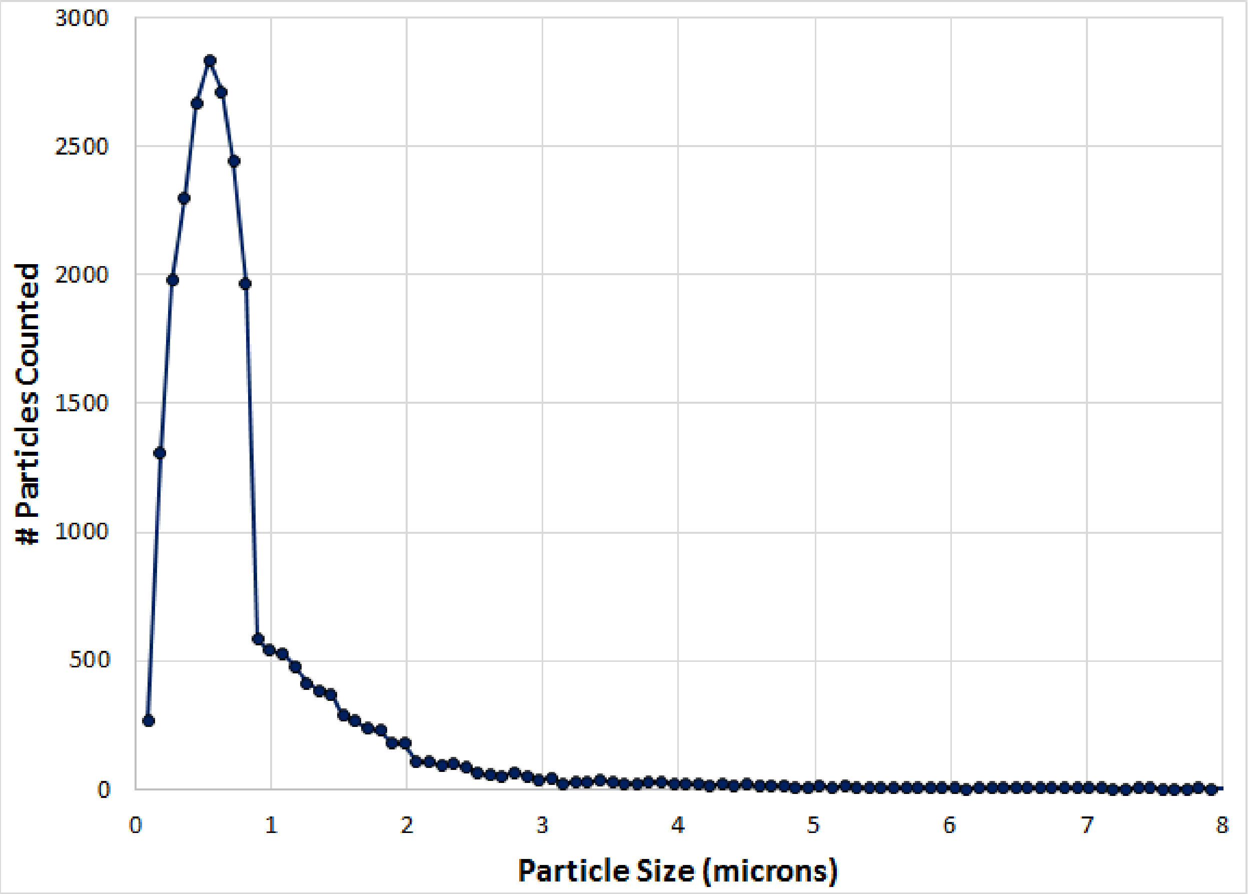

Figure S3: Results from another lab-settling experiment, using urban stormwater. Again, our mechanistic model better predicts PP removal over time compared to traditional first-order decay models with either high, medium, or low k values, which tend to either overshoot or undershoot the actual results.

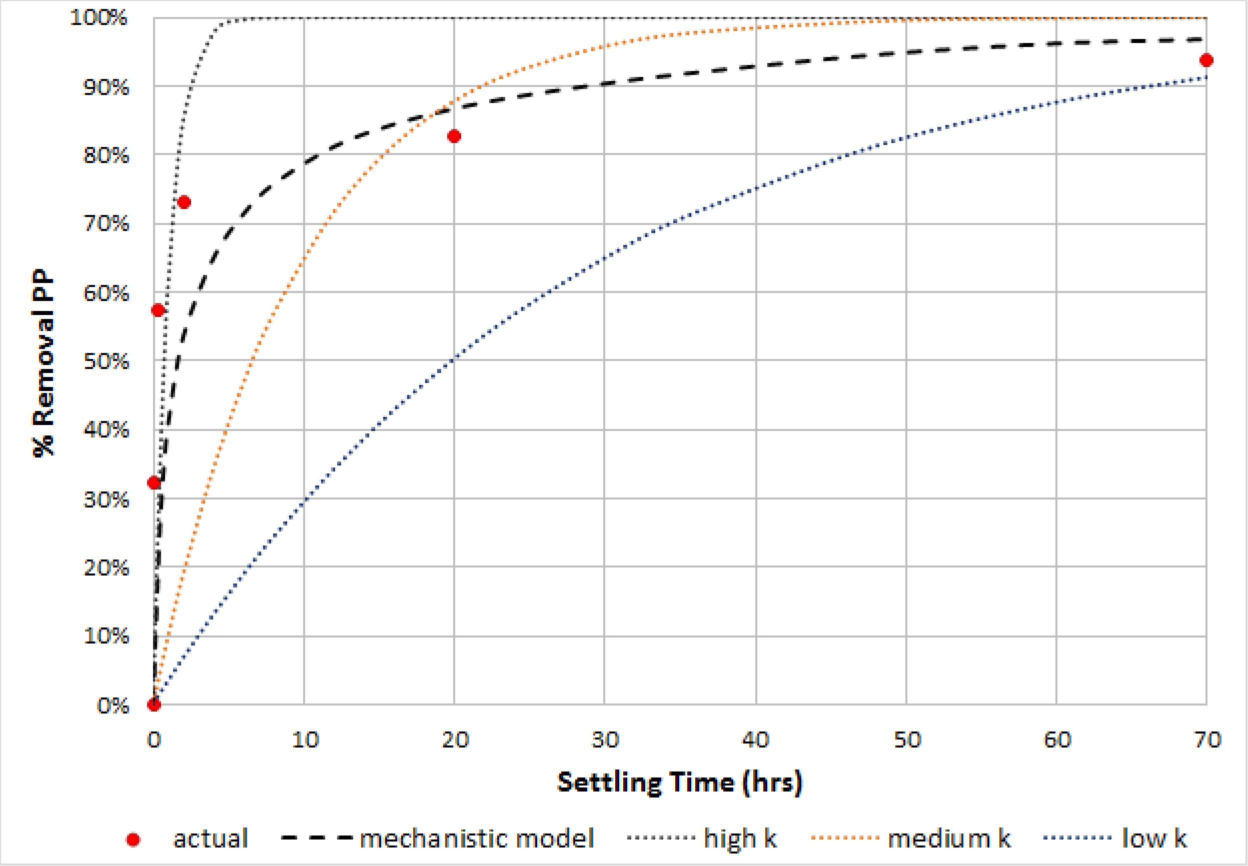

Figure S4: Typical scanning electron microscopy image of stormwater particles, used as an independent technique to verify the particle size distribution obtained via flow-imaging.

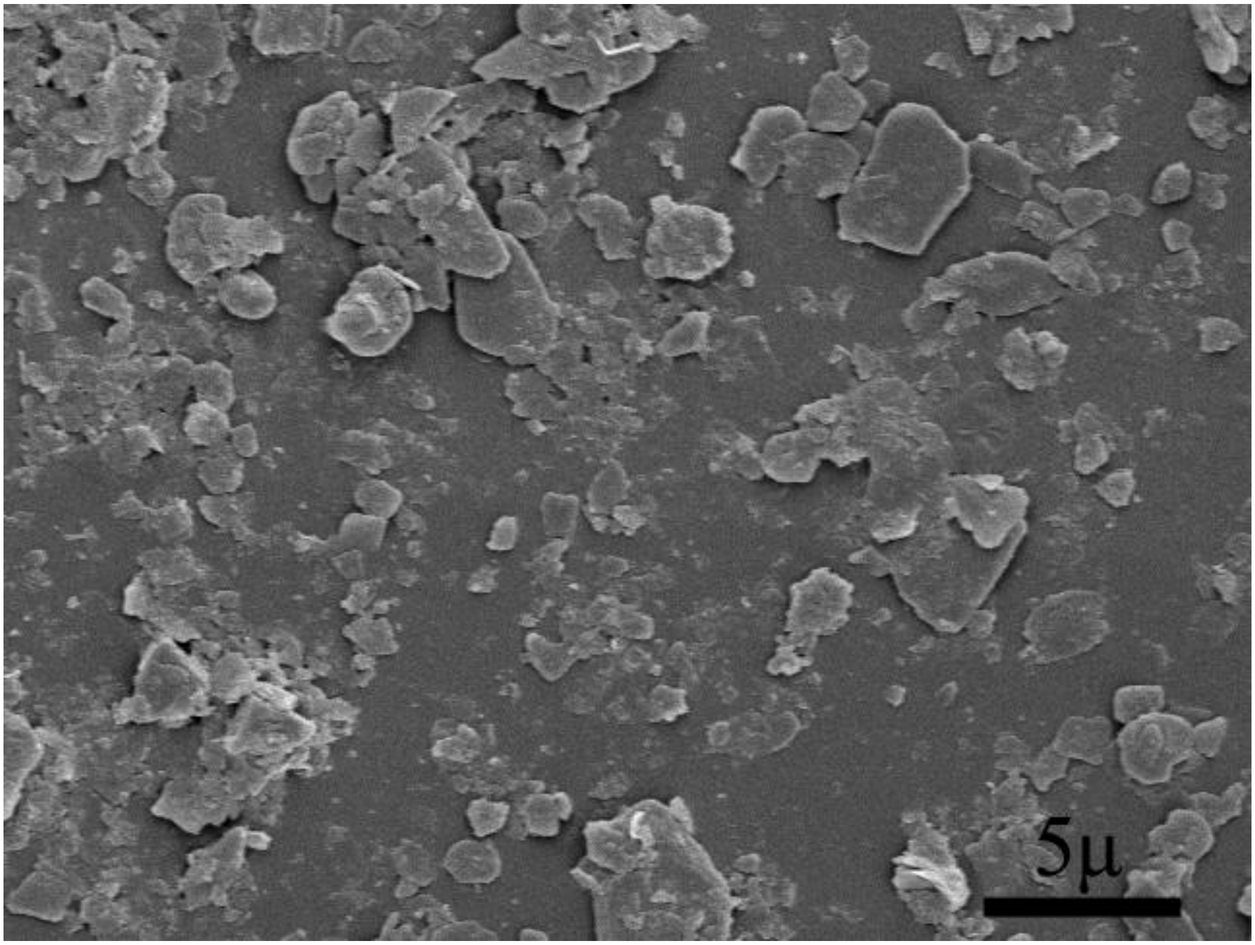

Supplement: Supplementary file 1 — Supplementary material 1 (PDF 232 kb) [file 13280_2017_981_MOESM1_ESM.pdf]
